# Phenotypic and Functional Characteristics of Blood Natural Killer Cells from Melanoma Patients at Different Clinical Stages

**DOI:** 10.1371/journal.pone.0076928

**Published:** 2013-10-18

**Authors:** Giulia Fregni, Meriem Messaoudene, Emmanuelle Fourmentraux-Neves, Sarra Mazouz-Dorval, Johan Chanal, Eve Maubec, Eduardo Marinho, Isabelle Scheer-Senyarich, Isabelle Cremer, Marie-Françoise Avril, Anne Caignard

**Affiliations:** 1 Cochin Institute, Institut National de la Sante et de la Recherche Medicale (INSERM) U1016, Centre National de la Recherche Scientifique Unité Mixte de Recherche 8104, University Paris Descartes, Paris, France; 2 Assistance Publique-Hôpitaux de Paris, University Paris Descartes, Department of Dermatology, Cochin Hospital, Paris, France; 3 Assistance Publique-Hôpitaux de Paris, University Paris Diderot, Department of Dermatology and Department of Pathology, Bichat Hospital, Paris, France; 4 Institut de Recherche des Cordeliers, Paris, France; University of Palermo, Italy

## Abstract

Melanomas are aggressive skin tumors characterized by high metastatic potential. Immunotherapy is a valuable alternative for metastatic melanoma patients resistant to chemotherapy. Natural Killer (NK) cells are efficient anti-tumor cytotoxic effectors. We previously showed that blood NK cells from stage IV metastatic melanoma patients display decreased NK receptors and that chemotherapy modifies the functional status of blood NK cells. To investigate the role of NK cells along melanoma progression, we have here studied NK cells from patients at different stages of the disease. First, we showed that *ex vivo* NK cells from certain stage III–IV patients displayed low degranulation potential. Using a dynamic label-free assay, we found that immunoselected IL-2 activated blood NK cells from patients efficiently lysed melanoma cells through NKp46 and NKG2D receptors, independently to the clinical stage. Moreover, the *ex vivo* phenotype of circulating NK cells from 33 patients (stage I to IV) was extensively analyzed. NK cells from patients displayed higher variability in the percentages of Natural Cytotoxicity Receptors (NCR) and Natural Killer Group 2D (NKG2D) receptor expression compared to donor NK cells. The main defect was the decreased expression of NCR1 (NKp46) by NK cells from metastatic patients. Interestingly, we found a positive correlation between the NK cell percentages of NKp46 and the duration of stage IV in melanoma patients. Finally, we showed that NK cells infiltrated primary melanomas and displayed a predominant peritumoral distribution. These results are new arguments for the development of NK-based therapies in melanoma patients.

## Introduction

Natural Killer (NK) cells are innate immune cells that have the natural ability to distinguish normal cells from “modified” cancer cells [1]. Activated NK cells eliminate their targets through the release of cytotoxic enzymes and produce soluble factors (chemokines and inflammatory cytokines) which in turn recruit and/or activate other effectors. Activating and inhibitory receptors present on NK cells are triggered during target cell recognition and the integration of these opposite signals determines NK activation [2], [3]. The main activating NK receptors are NKG2D and the natural cytotoxicity receptors (NCRs) NKp30 and NKp46. Activating and co-receptors recognize various ligands on targets usually upregulated upon cellular stress [4]. NK cells also express CD16, the low-affinity receptor for the Fc fragment of immunoglobulin which induces antibody-dependent cellular cytotoxicity (ADCC). Most inhibitory receptors recognize different HLA-class I molecules and include the killer immunoglobulin receptors (KIRs), NKG2A, CD85j, and LAIRs [5]–[7]. Accordingly, NK cells can kill target cells that have lost (or express low amounts of) HLA-class I molecules, frequent alterations seen in tumor cells including melanoma cells. However, cancer cells and tumor environment have the ability to escape from NK cell recognition through the modulation of NK activating receptors and/or by the secretion of immunosuppressive factors. NK cells infiltrate various tumors including melanoma [8] and their frequencies have been related to favorable clinical outcome [9]. A positive association has been reported between numbers of NK-TILs (anti-CD56 staining) and regression of melanocytic lesions [10].

Melanoma is a severe form of skin cancer due to its capacity to form metastases. Clinical and experimental arguments indicate that melanomas are immunogenic tumors. Cases of spontaneous regressions with the presence of large infiltrate of immune cells comforted the role of the immune system in tumor control [11], [12]. The characterization of numerous tumor antigens has allowed the development of antitumor immunotherapies during the last 15 years. However, the efficiency of the different strategies to amplify the specific T cell responses, *in situ*, was overall limited [13]. Whereas the immune system failure is well documented in advanced cancer patients, only few reports investigated immune alterations in early stage of tumor development.

We previously showed that before chemotherapy treatment, circulating NK cells from stage IV melanoma patients exhibited unique phenotype and function *ex vivo* compared to donor-derived NK cells [14] and that are modified by chemotherapy. We now investigate the presence of NK cells in primary melanoma tumors and the anti-melanoma NK function of circulating NK cells from stage I to IV patients. We show that IL-2 activated NK cells from patients efficiently lyse melanoma cells independently to their clinical stage. Interestingly, we identified NK cells infiltrating primary melanoma and we observed that the low expression of NKp46 in stage IV melanoma patients seemed to correlate with reduced stage IV duration.

## Materials and Methods

The study protocol and the consent procedure were approved by an ethic committee “Ile de France” (CPP: 2834) and the Declaration of Helsinki protocols were followed. All patients included in the present studies provided their written informed consent to participate to these studies.

### Collection of samples, PBMC isolation, NK cell immunoselection and cell lines

Blood samples (20–25 ml in EDTA collection tubes) from 33 melanoma patients at different clinical stages (Table S1) were obtained after written informed consent. All patients were studied before any chemotherapy. Blood samples from 10 healthy donors (HD) were analyzed as controls. PBMC were isolated by Ficoll-Paque PLUS (GE Healthcare Bio-Sciences AB) density gradient centrifugation. For xCELLigence assay, donor and patient NK cells were purified by negative immunoselection using the NK selection kit (Miltenyi Biotech). Purified NK cells (0.5–1×10^6^/mL) were cultured in RPMI 1640 medium (GIBCO Invitrogen) supplemented with IL-2 (10 ng/mL; Miltenyi Biotech) and 10% human serum AB (Biowest) for 6 days.

The MelC cell line previously isolated from a metastatic lymph node of a stage III melanoma patient [14] and K562 cells (0.3 to 1×10^6^/ml) were maintained in RPMI 1640 supplemented with 10% FCS (Biowest) and 1% Penicillin-Streptomycin (Invitrogen).

### CD107a degranulation and IFNγsecretion assays

In brief, 10^6^ PBMC were cultured at 10∶1 effector/target ratio for 4 h at 37°C with K562 cells in V-bottom plates. Cells were then labeled for 30 min at 4°C with anti-CD16-PeCy7 (Beckman Coulter), anti-CD56-HorizonV450, anti-CD3-APC-H7, anti-CD107a-FITC (BD Biosciences) and anti-IFNγ-APC (MiltenyiBiotech), fixed with 2% paraformaldehyde in PBS and collected on a FACSCantoII flow cytometer. Results are expressed as the percentage of IFNγ^+^ and CD107a^+^ NK-gated cells. Spontaneous NK IFNγ secretion and degranulation (CD107a percentages) were determined in absence of targets.

### xCELLigence assays

NK-mediated lysis of MelC cells was assessed by xCELLigence, a label-free, real-time monitoring assay of adherent cell lysis by measuring impedance [15]. We previously showed that this assay correlated with degranulation test [16]. Briefly, MelC adherent targets (15,000 cells) were seeded into the wells of 96× E-Plates in 100 γL of media and their adhesion was monitored for 5 hours. IL-2-activated NK cells were added in a volume of 50 γL/wells. Co-cultures were assessed by the system with a measure of cell index every 30 minutes for up to 300 min. The co-cultures were carried out at 2∶1 E/T ratio. In some co-cultures, anti-NKp46, anti-NKp30, anti-NKG2D or anti-DNAM1 mAbs (R & D System) were added with NK cells. Results are expressed as lysis percentages determined as previously described [16].

### Flow cytometry analyses

PBMC were resuspended in PBS1× supplemented with 5% human serum AB (Biowest) and incubated 30 min in ice to block nonspecific FcR binding. Cells were then washed and stained 30 min at 4°C with the mAbs diluted in PBS1×/FCS2%/EDTA2mM at predetermined optimal concentrations. NK cells, gated on CD3^−^CD56^+^ cells in lymphocyte FSC/SSC subset (BD Pharmingen) were analyzed for the expression of NKp46, NKp30, NKp44, NKG2D (PE-conjugated antibodies, BD Pharmingen), CD16 (PE-Cy7-conjugated antibodies, Beckman Coulter), HLA-DR and CD69 (FITC-conjugated mAb, Miltenyi Biotech) after dead-cell and doublet exclusion. The percentages of positive cells were determined on more than 3000 NK-gated events. Cells were collected on a FACSCantoII flow cytometer (BD Biosciences) and data analyzed with BD FACSDiva software. Electronic compensation was set up using BD CompBeads anti-mouse (BD Biosciences).

### Immunohistochemistry and NK cell quantification

Paraffin-embedded tumors were retrieved retrospectively from 9 patients diagnosed with localized melanoma (<2 mm in Breslow's thickness) at Bichat Hospital (Paris, France). The distribution of melanoma-infiltrating NK cells was assessed by immunohistochemistry using anti-NKp46 mAb (R&D systems) as previously described [17]. After staining, whole tissue sections were scanned using NanoZoomer (Hamamatsu Photonics). NKp46-positive NK cells were then counted using NDP. View software in 10 fields of 1 mm^2^, in the center of the tumor and in the peritumoral areas.

### Statistical analysis

Statistical tests and graphics were generated by Prism version 5 (GraphPad Software Inc, LaJolla, CA, USA). The non-parametric Kruskal-Wallis (K-W) test was used to compare the medians of NK proportions, the percentages of NK receptors and CD107a and cytokine production by NK cells between different groups (p values≤0.05 noted as *, p<0.01 as ** and p<0.001 as***). Comparisons between spontaneous and K562-induced CD107a and INFγ production by NK cells were analyzed with the paired *t*-test. Comparisons between two groups were assessed by non-parametric Mann-Whitney test. Correlations between the percentages of NK receptor expression were analyzed by Pearson test. The survival curves were plotted according to the Kaplan-Meier method and compared using the Log-rank test.

## Results

### Patient characteristics and NK cell proportions in melanoma patients

We prospectively collected blood samples from 33 patients with localized to metastatic disease. Based on the melanoma classification that takes into account the Breslow index, the presence of ulceration, the involvement of regional lymph nodes and the presence of metastasis, patients were stratified into three groups according to their clinical stage (Table 1): stage I–II (n = 7), stage III (n = 15) and stage IV (n = 11). Stage I–II patients had localized melanoma without involvement of regional lymph nodes. Stage III patients displayed metastatic regional lymph nodes without distant metastases. Stage IV patients had distant metastases restricted to lymph nodes for M1a patients, lung metastases for M1b patients and other distant metastases for M1c patients. Healthy donors (n = 10) were included as controls. Stage I to III patients received no adjuvant therapy and in particular no chemotherapy and stage IV patients were analyzed before chemotherapy. Only few patients had a lymphopenia (<1000/mm^3^): 4/15 stage III. LDH values were increased (>400 UI) in 4/15 stage III and 3/11 stage IV patients (Table S1).

**Table 1:** Patient characteristics.

	Stage I-II	Stage III	Stage IV
**Gendre:** M/F	5M/2F	5M/10F	5M/4F
**Age:** years (min-max)	61 (55–71)	65,7 (39–90)	56,1 (22–81)
**Lymphocytes counts:** ly/mm^3^ (min-max)	2500 (1376–3900)	1572 (600–2730)	1926 (1070–4283)
**LDH:** mU/ml (min/max)	NA	370 (270–465)	464 (288–912)
**Subtype TNM classification**			
**A**	IA:1; IIA:1	1	**M1a:** 3
**B**	4	5	**M1b:** 2
**C**	1	9	**M1c:** 4

M: Male; F: Female; NA: Not Available.

**Figure 1:**
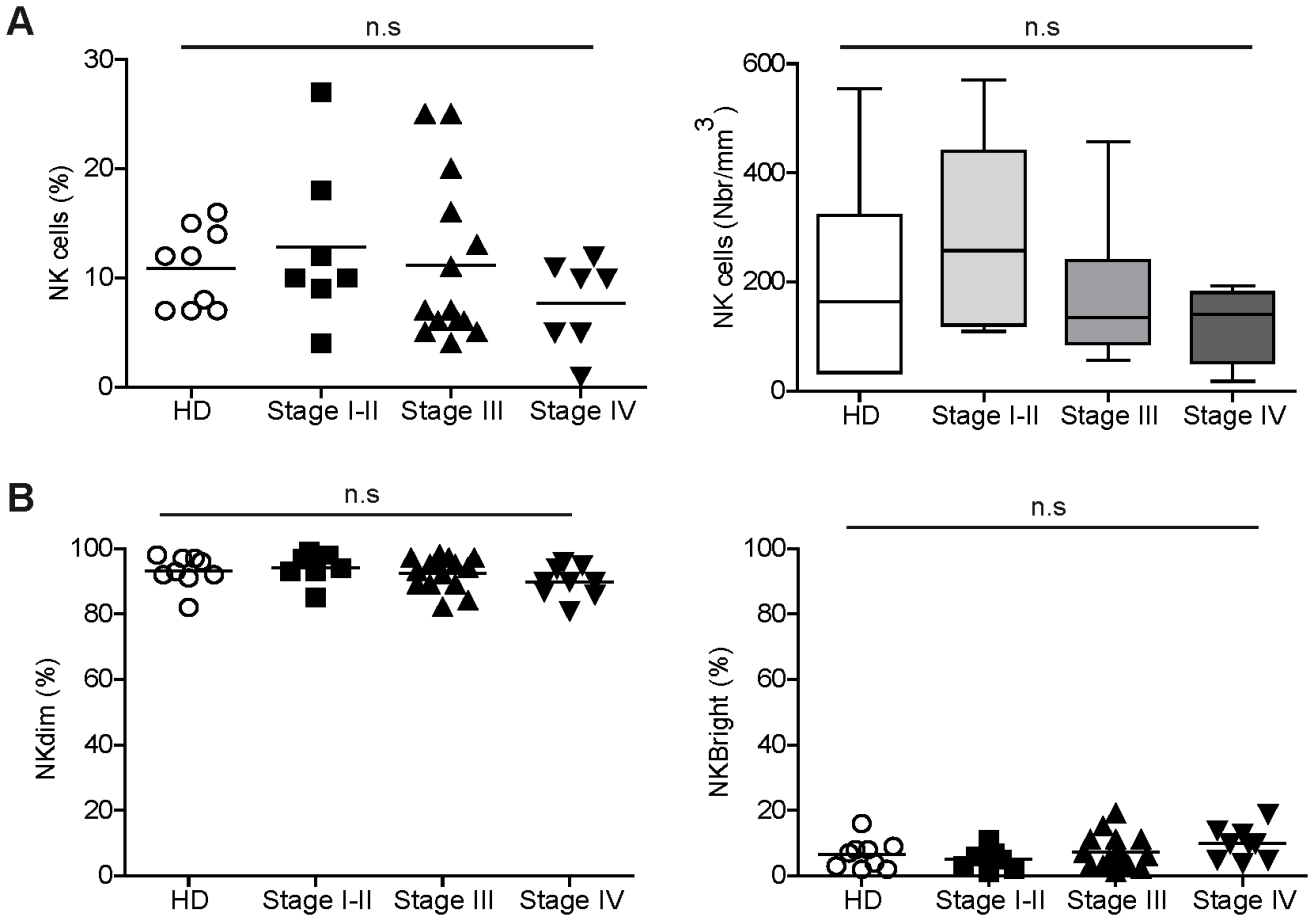
Analysis of blood NK cells from donors and melanoma patients. (A) Proportions (left) and absolute numbers (right) of NK cells among PBMC from donors and melanoma patients stratified according to their clinical stages. (B) Proportions of CD56^dim^ and CD56^bright^ cells among the NK cell population in donors and patients. Statistical analysis was assessed by non-parametric Kruskal-Wallis (K-W) test (n.s = not significant).

The proportions of circulating NK cells among the lymphocyte population were comparable between donors and patients (see gating strategy in Fig. S1). Considering the whole series of patients, the absolute numbers of NK cells in patients were in the range of those of donors as previously described [18]. However, some stage I–III patients displayed high percentages of NK cells while a trend for lower NK cell percentages and absolute numbers was observed in stage IV patients (Fig. 1A). Moreover, proportions of CD56^dim^ and CD56^bright^ NK cell subsets were similar in donors and patients (Fig. 1B).

### Function of circulating NK cells along disease progression

**Figure 2:**
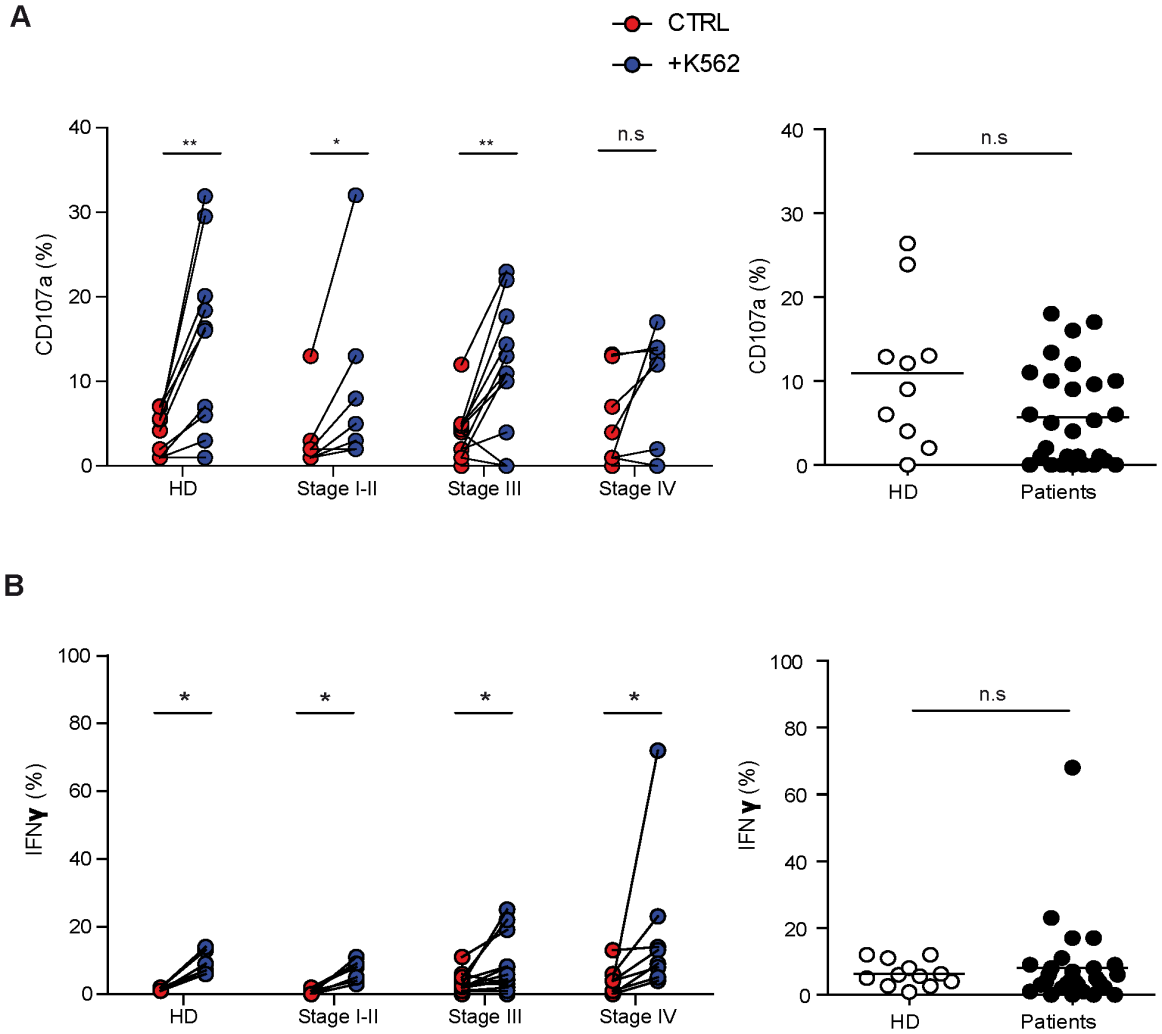
*Ex vivo* NK cell functionality in donors and melanoma patients. Patients were stratified (left panels) or not (right panels) according to their clinical stage. Freshly isolated PBMC were stimulated with K562 (E/T ratio 10/1 for 4 hours) in absence of cytokine and gated CD3^−^CD56^+^ NK cells were analyzed by flow cytometry. (A) Percentages of NK cell degranulation and (B) IFNγ production were assessed. On left panels, spontaneous (red) and K562-activated (blue) values are indicated. On right panels, the effective NK cell activation was reported calculated as K562-induced minus spontaneous. Comparisons between spontaneous (ctrl) and K562 induced activation of NK cells were analyzed with the paired *t*-test (p values≤0.05 noted as * and p<0.01 as **). Comparison of NK function between donors and patients (right panels) using the non parametric Mann-Withney test (n.s = not significant).

We first assessed the NK cell function by the analysis of the cytolytic potential (externalization of CD107a) and IFNγ production in response to K562 targets (Fig. 2). Freshly isolated PBMC were stimulated for 4 hours with K562 in absence of cytokine and gated CD3^−^CD56^+^ NK cells were analyzed. After activation, the percentages of CD107a were highly variable and some individuals displayed values close to spontaneous degranulation (Fig. 2A). No significant difference was observed in the levels of *ex vivo* degranulation between donors and patients (Fig. 2A, right panel). However, compared to donors or to patients with a local disease, more individuals characterized by non-degranulating NK cells were found in the groups of late melanoma stages (Fig. 2A). In particular, NK cells from only 1/10 donors and 1/7 stage I–II patients did not degranulate, compared to 4/13 stage III and 2/8 stage IV melanoma patients. Stimulation with K562 induced comparable levels of IFNγ production by patient and donor NK cells (Fig. 2B).

**Figure 3:**
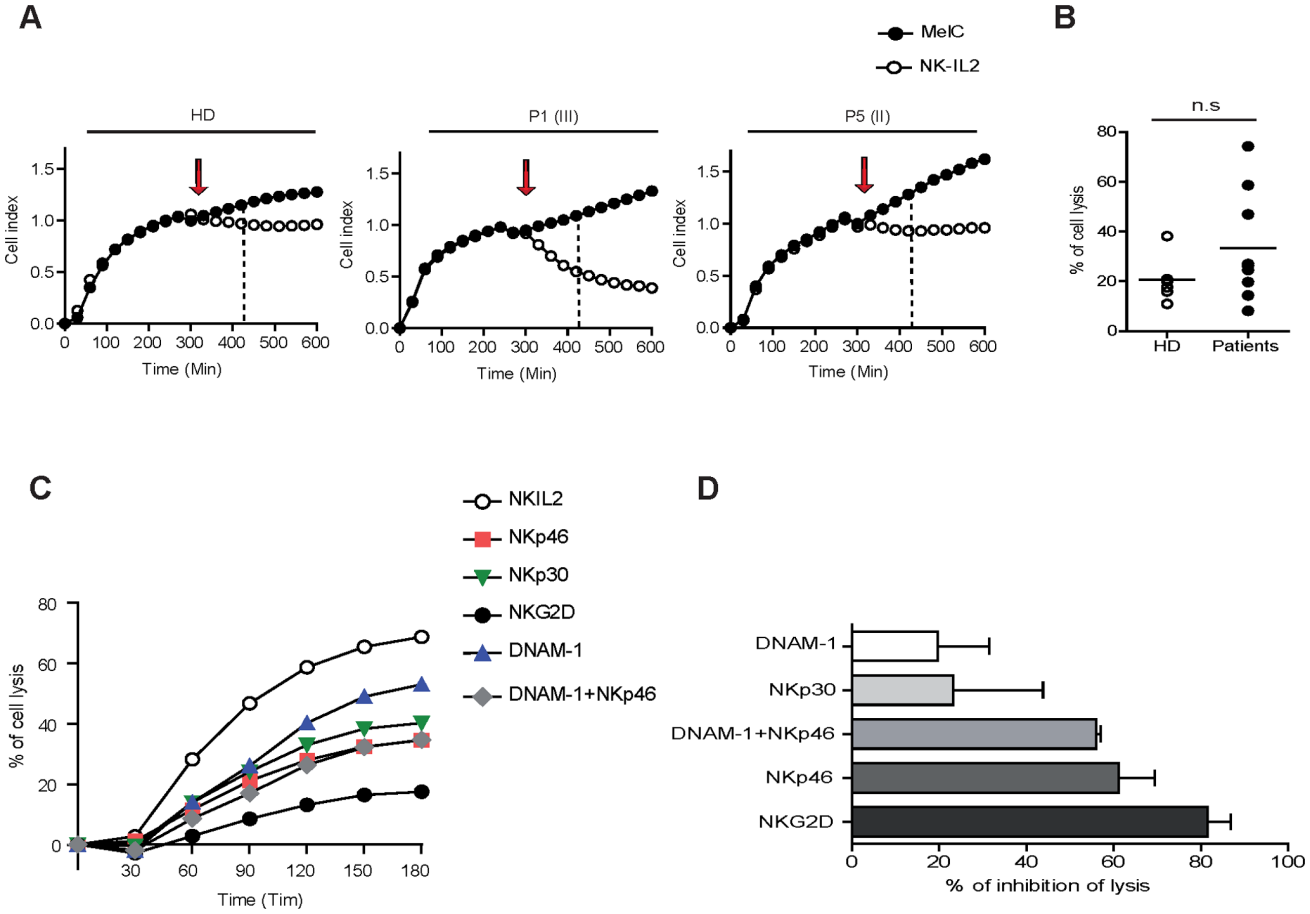
IL-2-activated NK cells from patients exhibited antitumor capacities. (A) Dynamic measure of MelC cell index (CI) alone (black dots) and in presence of immunoselected IL-2-activated NK (white dots) from 1 donor and 2 patients are shown. Targets are monitored for cell adhesion for 5 h before and after addition of NK cells (arrow). NK cells (ratio 2/1) induce a rapid decrease of adherent target cell index (dotted line: 120 min after NK addition). In brackets, the clinical stage of each patient was noted. (B) Percentages of melanoma cell lysis by NK cells from 9 patients and 6 donors calculated from CI curves after 120 min of NK/target interactions. (C) Cytolysis experiments (n = 5) were performed in presence of anti-NKp46, anti-NKp30, anti-NKG2D or anti-DNAM-1 mAbs alone or in combination. Cytolysis curves (percentages of lysis) in presence of blocking mAbs are depicted for one representative experiment. (D) The percentages of lysis inhibition induced by the blocking of NK receptors are calculated at 120 min of co-culture (mean values and SD of 5 independent experiments).

We then assessed the lysis of melanoma cells (MelC) by donor and patient NK cells, using a dynamic assay that measures the cell index of adherent targets [16]. Blood NK cells from 9 patients (stage II to IV) and 6 donors were immunoselected and activated by IL-2 for 6 days before measuring their capacity to lyse melanoma cells. The addition of NK cells to adherent melanoma cells induced a rapid decline of cell index that was proportional to lysis of targets (Fig. 3A). Percentages of MelC lysis at 120 min after NK cell addition are depicted in Fig. 3B. Donor NK cells exhibited low lytic capacities with limited inter-individual variability. IL-2 activated NK cells from patients efficiently lysed melanoma cells and displayed higher inter-individual variability than donors. The patient NK lytic activities were independent of the disease stage. It is interesting to note that while NK cells from only 1/6 donors lysed more than 25% MelC cells, NK cells from 5/9 patients induced a lysis higher than 20%. In particular, NK cells from one patient reached >75% lysis after 120 minutes of co-culture. Thus, IL-2 activated NK cells from patients acquired high lytic potential towards metastatic lymph node melanoma cells.

In addition, in 5 independent experiments using NK cells from stage II to IV patients, we repeated the co-cultures in presence of mAbs blocking activating receptors (Fig. 3C and D). Addition of anti-NKp46 or anti-NKG2D mAbs strongly decreased melanoma cell lysis while the inhibition induced by anti-NKp30 mAbs was lower. Addition of anti-DNAM-1 mAbs alone exerted a modest effect and displayed no potentiating effect when combined with NKp46 (Fig. 3C and D). These data indicate that NKp46 and NKG2D receptors are involved in the lysis of melanoma cells by IL-2 activated NK cells.

### Expression of NK receptors by circulating NK cells from melanoma patients at the different stages of disease

**Figure 4:**
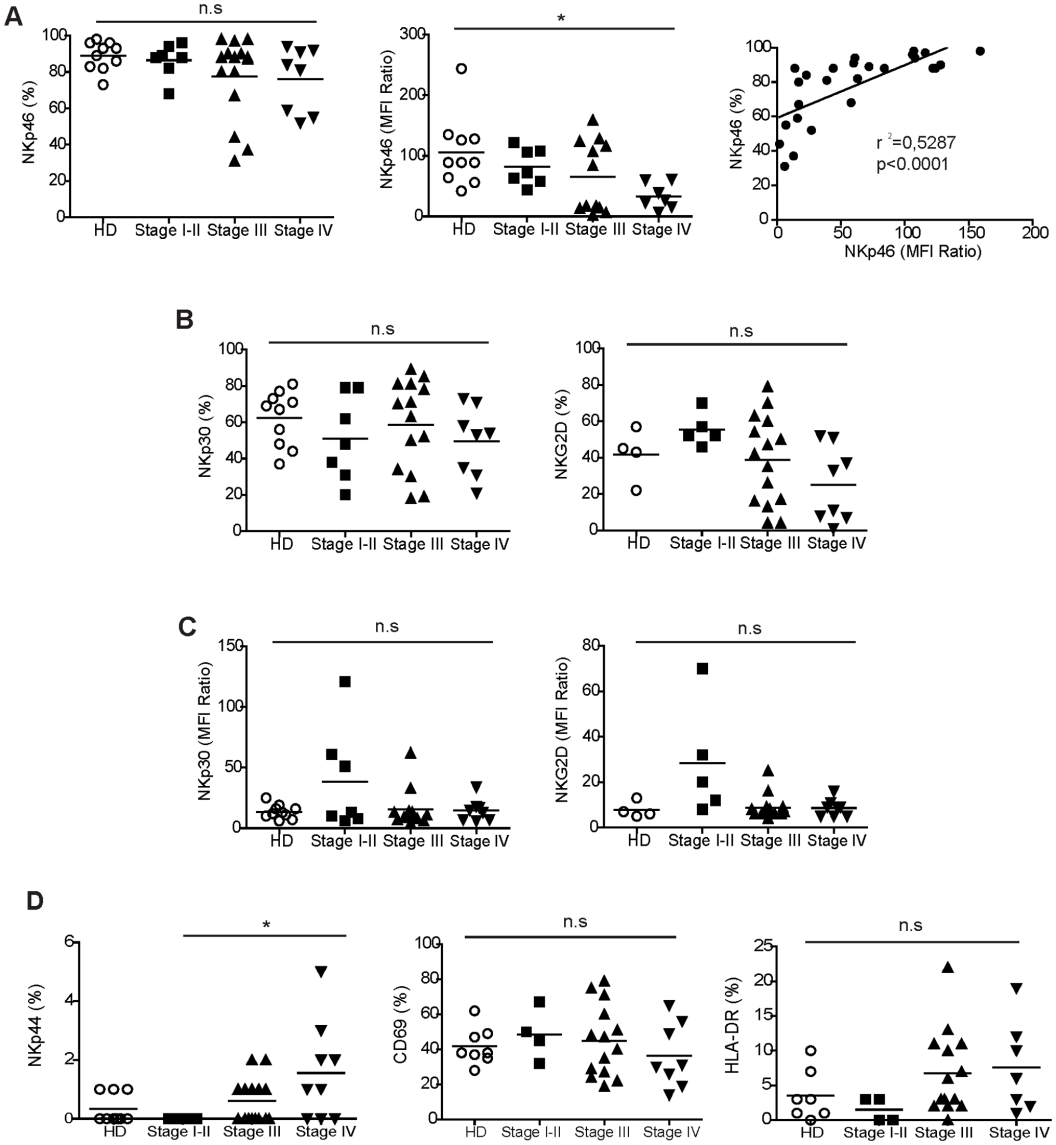
Phenotype of circulating NK cells from donors and patients along melanoma progression. (A) Percentages (left) and levels of expression (MFI Ratio; middle) of NKp46 receptor by NK cells from donors and patients at different stages of disease; on the right, the correlation between NKp46 percentage and MFI ratio in patients. (B) Percentages of NK cells expressing NKp30 and NKG2D receptors in melanoma patients. (C) Levels of expression (MFI ratio) of NKp30 and NKG2D in melanoma patients. (D) Percentages of expression of NKp44 receptor and CD69 and HLA-DR activation molecules. Comparisons between the different groups were analyzed with the non-parametric K-W test (n.s = not significant; * p<0.05). Correlations were analyzed with Pearson test. Regression lines (r^2^) and p-values are reported.

From the same cohort of patients, we extensively studied *ex vivo* the phenotype of NK cells by multicolor flow cytometry on gated CD3^−^CD56^+^ cells from PBMC. We observed a higher variability in the percentages of activating receptor expressed by patient NK cells compared to donor NK cells (Fig. 4). We previously showed that NK46 expression (percentages and expression level: MFI ratio) was decreased in stage IV patients compared to donors and that it was augmented after chemotherapy. In the present studies, we found that the percentages and MFI ratios of NKp46 in patients progressively decreased with advanced disease: the MFI ratios were significantly lower in stage IV patients compared to donors. Moreover, the percentages and the expression level of NKp46 were highly correlated (Fig. 4A). The expression of NKp30 and NKG2D (% and MFI ratios) were comparable in donors and patients. The percentages and MFI ratios of NKp30 and NKG2D were also more variable in patients with some individuals characterized by low percentage values and MFI ratio (Fig. 4B, C). Surprisingly, we observed that NKp44 was expressed by NK cells from certain stage III (7/15) and IV (6/9) melanoma patients (Fig. 4D). Finally, we analyzed the expression of CD69 and HLA-DR activation markers (Fig. 4D). CD69 was expressed at similar MFI ratios (not shown) by 30–40% of NK cells in donors and patients. The expression of HLA-DR was also comparable between donors and the groups of patients.

### NKp46 correlation with stage IV duration

**Figure 5:**
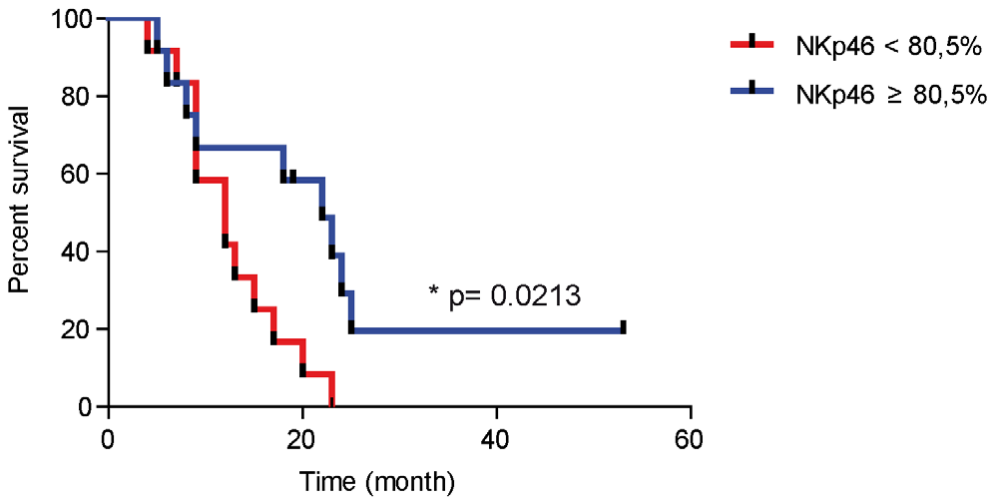
Prognostic values of NK cell parameters in the natural course of melanoma. Kaplan Meier curves were designed to analyze the association between NKp46 expression by NK cells and duration of stage IV. Patients (n = 24) were divided into two subsets of 12 patients defined by a cut off of NKp46 expression corresponding to the percentage median value (80.5%; p value<0.5 noted as *).

The main alteration that we observed in NK phenotype concerns the decreased expression of NKp46 receptor in stage III and IV melanoma patients. To assess the relevance of this observation, in a cohort of 24 stage IV patients, we analyzed the expression of this receptor in correlation with the clinical evolution of patients. The duration of stage IV was used as a parameter of the disease evolution. In the whole stage IV patient series, we calculated the median percentage value of NKp46 to design Kaplan-Meier curves and determine the relation between NKp46 expression and duration of stage IV. Surprisingly, we found a positive correlation between the NKp46 expression (%) and the duration of stage IV (Fig. 5).

### NK cells infiltrate primary melanomas

**Figure 6:**
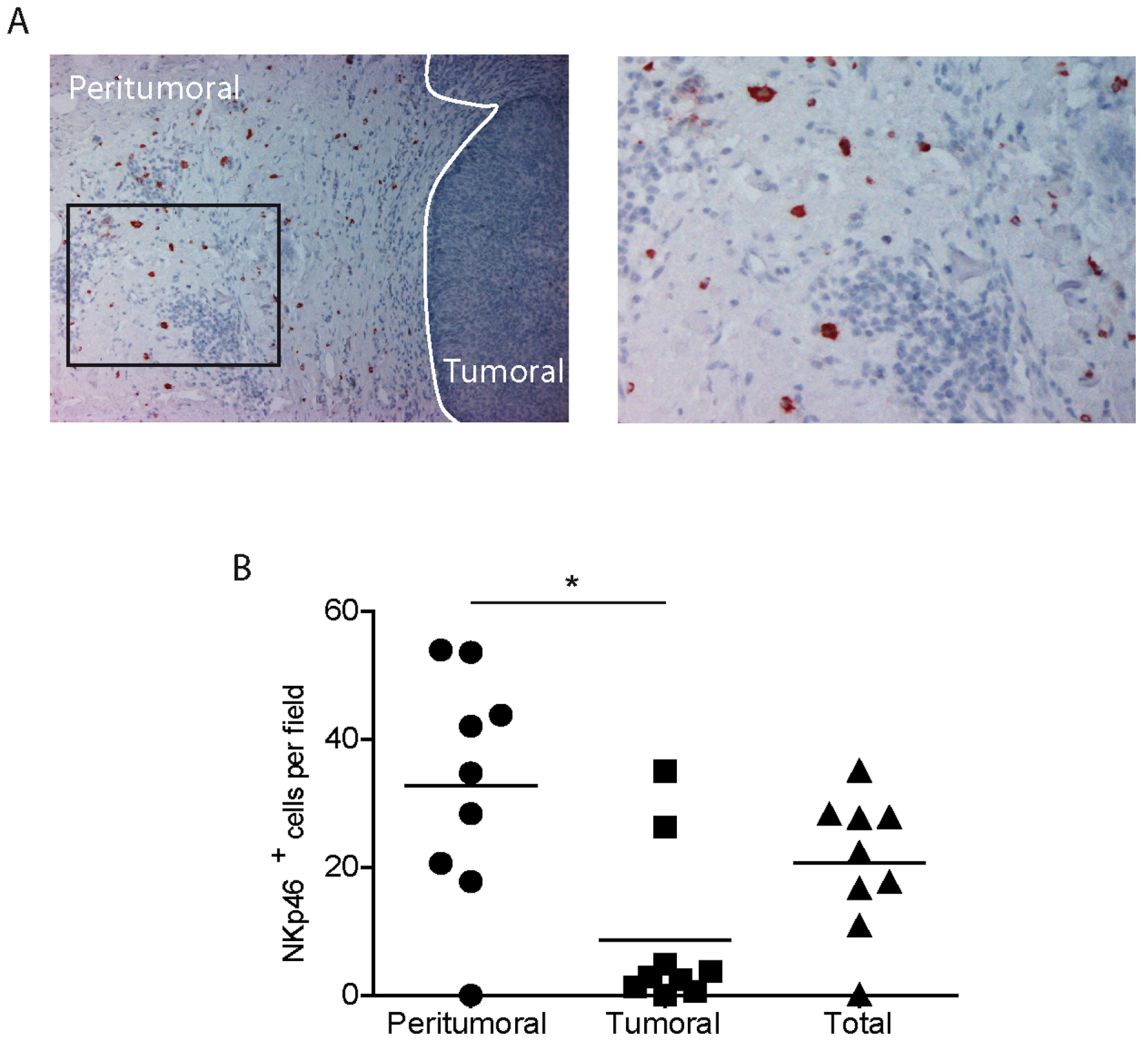
NK cells are present in primary skin melanoma microenvironment. The presence of NK cells (in red) was analyzed in paraffin-embedded thin melanoma sections by anti-NKp46 immunohistochemical labeling. (A) One representative image is reported. Original magnifications: ×10 (left panel) and ×20 (right panel) followed by computer magnification. (B) NKp46^+^ cells were counted in 10 tumoral and 10 peritumoral fields as described in material and methods. Results are expressed as mean values. Horizontal lines represent median values. NK cell numbers were compared with paired t test (p = 0.048).

Nine primary melanomas were analyzed by immunohistochemistry (IHC) to determine the frequency and distribution of infiltrating NK cells. Traditionally, NK cells are defined as CD3^−^CD56^+^CD16^+/−^ and are difficult to detect *in situ* by a unique marker since melanoma cells also express CD56. Here, we have studied melanoma-infiltrating NK cells by anti-NKp46 antibody staining, as recently described for lung cancers [17]. One representative tumor is shown in Figure 6A and the others are depicted in Figure S2. NKp46^+^ cells were counted in 10 fields of 1 mm^2^ each and the average was calculated. A median value of 20 NKp46^+^ cells per field was determined when the whole sample was analyzed. However, the distribution was not equal between peritumoral and tumoral areas and NK cells are preferentially localized in the periphery of the tumor (Fig. 6B). In the peritumoral areas, large granular NKp46^+^ cells with membrane and cytoplasmic staining are abundant in certain tumors close to blood capillaries. In tumor foci, rare large and granular NKp46 cells are found (Fig. 6A).

## Discussion

Innate immunity effectors, NK cells are likely involved in the immune response against melanoma. An accurate understanding of how they interfere with tumor development and progression is required for drawing up efficient NK cell-based immunotherapies.

In order to get new insight on NK cell involvement in melanoma progression, we first analyzed the stage dependent changes in the expression of activating NK cell receptors in melanoma patients. We have previously shown that pre-chemotherapy NK cells from metastatic stage IV melanoma patients display a reduced expression level of NKp46. In the present studies we confirm the reduced expression of NCR (NKp46) in some stage III and IV melanoma patients. Certain advanced patients also exhibited low expressions of NKG2D and NKp30 receptors. While the expression of activating NK receptors (NKp46, NKG2D, NKp30) was down-regulated in certain patients, NK cells from advanced disease expressed activation molecules (CD69, HLA-DR) and displayed NKp44 induction (Fig. 4D). These data suggest that NK cells from metastatic patients are terminally mature NK cells endowed with a particular activated status despite reduced NKp46 expression.

Compared to donors, a decreased expression of the activating NKG2D receptor and an increased expression of the inhibitory KIR, CD158b, were described on NK cells from metastatic melanoma patients and correlated with an impaired NK cell function [19]. The combined CD161^low^/NKG2D^low^ expression was reported and correlated with the altered cytokine-induced NK cell activity in metastatic melanoma patients [20], [21]. NCR and NKG2A were not analyzed in these reports and the eventual treatment of patients enrolled in these studies was not indicated.

According to the tumor type, the regulation of NK cells along disease progression may largely differ. In breast cancers, the expression levels of NKp30, NKG2D, DNAM-1, 2B4 and CD16 were lower in patients with invasive cancers than in patients with *in situ* tumors and in control group. In contrast, two inhibitory receptors, NKG2A and CD85j, were upregulated in the metastatic group compared with control and *in situ* tumor groups. Thus, the concomitant underexpression of activating receptors and overexpression of inhibitory receptors in invasive tumors correlated with severely decreased NK function in metastatic patients [22]. These results indicate that while circulating NK cells exhibit functional defects in most patients with advanced cancers, the severity of these deficiencies depends on the tumor type [23].

We have studied the *in situ* distribution of NK cells in primary melanoma. The presence of NK cells infiltrating melanoma has rarely been analyzed, likely because of the technical difficulties for accurate NK cell identification by single staining. Previous studies reported that NK cells were rare in primary melanoma (1/8) and dysplasic nevi (3/31) but were abundant in metastases [8]. CD56^+^ NK cells studied in 18 primary melanomas represented 1% of the TIL [24]. CD56^+^ NK cells (but not T cells) were more frequent in regressing melanoma compared to nevi with regression and non regressing melanoma [10]. In contrast, a recent study by tissue microarray reported that tumor infiltrating NK cells are rare in melanoma metastases [25]. The different mAbs and technical devices used to stain NK cells in tissue may account for these discrepancies. In the present study, we assessed the *in situ* distribution of NK cells in primary melanomas using a mAb specific for NKp46. We found that NK cells are abundant in the peritumoral tissue and rarely infiltrate tumor nests.

The presence of NKp46^+^ NK cells infiltrating primary melanoma, the reduced expression of NKp46 by NK cells in certain stage IV patients and its likely correlation with the duration of stage IV are new arguments for the importance of this receptor in the innate immune response against melanoma. The role of NKp46 in the response to tumor was already reported. NKp46 labels NK cells *in situ* and is a useful marker for correlation studies between immune cell infiltrates and tumor prognosis/evolution. Thus, it was shown that high NK cell densities are associated with improved survival in lung metastases from renal tumors, not from colon rectal tumors [26]. In gastro-intestinal sarcomas (GIST), infiltration by CD56^bright^ NK cells of tumor foci predicted response to treatment by Imatinib [27]. NKp46 was shown to control melanoma metastases in mouse model [28], tumor growth of lymphoma tumors [29]. Staining with chimeric NKp46Fc indicated that NKp46 ligand expression correlated with malignant potential of the lesion [30].

The tumor and its microenvironment can modulate the development and maturation of NK cells along the disease progression [31]–[33]. Although melanoma cells express stress molecules and frequent alterations of HLA-I molecule expression favoring NK cell activation [34], they also secrete immunosuppressive factors (TGFβ, IDO, PgE2) altering the expression of several activating NK receptors (NKp30, NKp44, NKG2D) [35], [36]. Membrane and soluble HLA-E molecules can also contribute to cancer immune escape by their interaction with the inhibitory receptor NKG2A on NK and T cells; their immunosuppressive activity yet unknown is likely [37]. We previously reported a positive correlation between the presence of seric HLA-E molecules and melanoma [38]. Here, we found a trend towards increased concentrations of soluble HLA-E molecules in the sera of stage IV melanoma patients (data not shown). We also described low seric concentration of IL-12 in some patients (data not shown). Altered DC and/or macrophage maturation may explain the defect of IL-12 [39]. Alternatively, tumor-derived MMP2 may prime DC for Th2 cytokine response [40]. IL-12 is required for the optimal NK cell activation by dendritic cells [41] and favors helper NK cell activity for melanoma CTL induction [42]. Changes in the seric concentrations of IL-12 and soluble immunoregulatory HLA-E molecules (data not shown) may contribute to the modulation of NK cells in metastatic patients.

Our data represent original additional findings on the role of NK cells in the immune response against melanoma along disease progression. In patients, NK cells exhibit phenotypic defects that may alter their function *in vivo* but these cells remain responsive to IL-2 activation, acquiring high lytic capacities towards melanoma cells (Fig. 3).

NK-based therapies may have interest in the context of the actual development of MAP-Kinase inhibitors for the treatment of melanoma patients bearing B-RAF mutations. B-RAF inhibitors emerged as a valuable treatment for melanoma patients inducing rapid responses, improving overall survival and progression free survival but responses lack durability [43]. The present data are arguments for combining MAP-Kinase inhibitors with NK-based immunotherapy with the aim to induce prolonged clinical responses.

## Supporting Information

Figure S1 Gating strategy of NK cells in total LN cell suspension. First two panels show SSC and FCS plots with elimination of doublets. Third panel shows SSC/FSC plot with the lymphocyte gate, followed by SSC CD3 plot (Fourth panel). Panel 5, NK cells were identified with CD56 and CD16 staining.
(TIFF)

Figure S2 In situ detection of NK cells. Anti-NKp46 staining in 8 primary skin melanoma microenvironment (Red-brown cells) (A-H). Original magnification: ×10 (left panel) and ×20 (right panel) followed by computer magnification.
(TIFF)

Table S1 Patient and tumor characteristics
(TIFF)
