# Supplementary figures and images for: Phenotypic and Functional Characteristics of Blood Natural Killer Cells from Melanoma Patients at Different Clinical Stages
Source: PLoS One. 2013 Oct 18;8(10):e76928. doi: 10.1371/journal.pone.0076928 (PMC3799851; doi:10.1371/journal.pone.0076928)

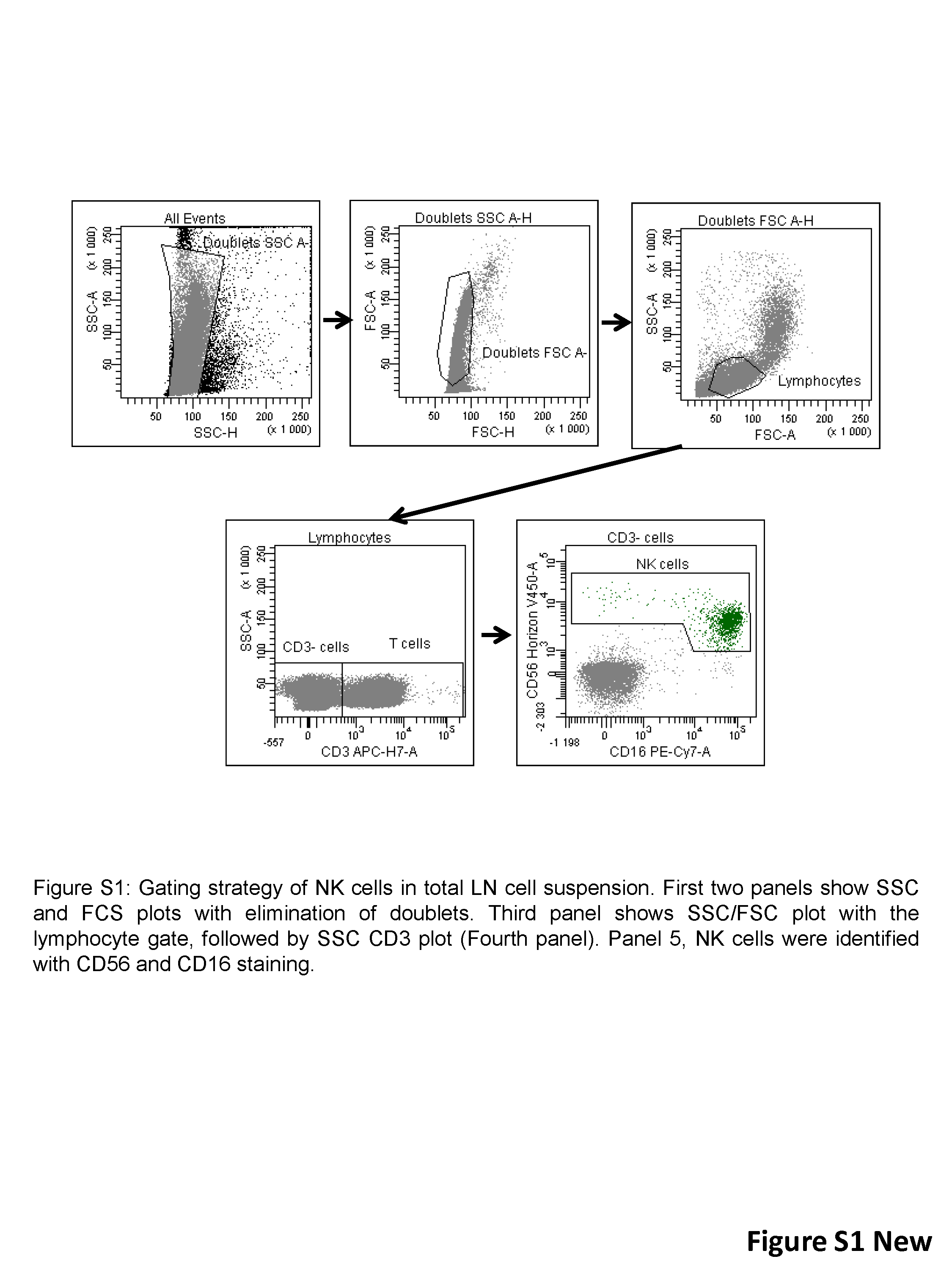

Supplement: Figure S1 — Gating strategy of NK cells in total LN cell suspension. First two panels show SSC and FCS plots with elimination of doublets. Third panel shows SSC/FSC plot with the lymphocyte gate, followed by SSC CD3 plot (Fourth panel). Panel 5, NK cells were identified with CD56 and CD16 staining. (TIFF) [file pone.0076928.s001.tiff]

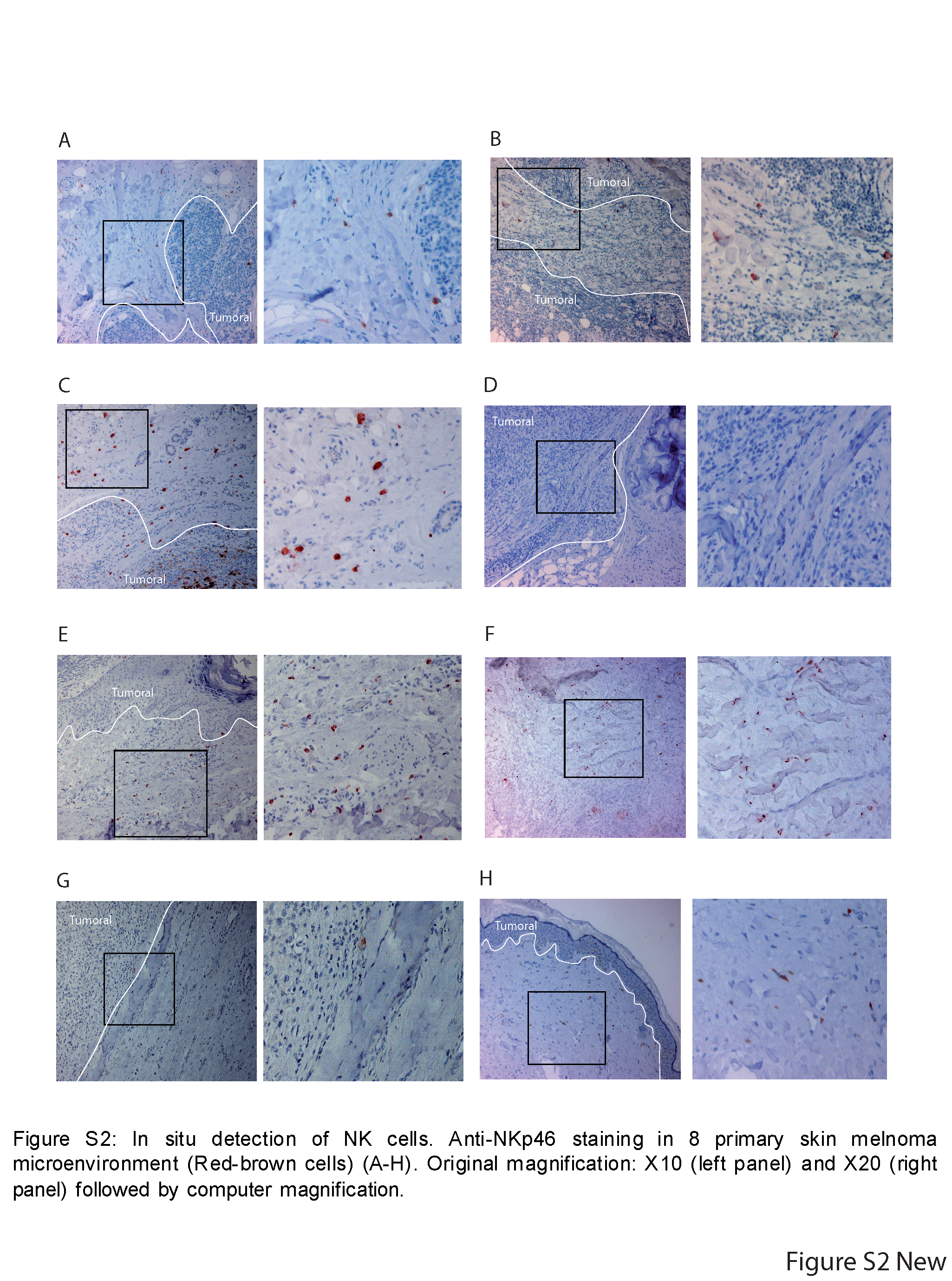

Supplement: Figure S2 — In situ detection of NK cells. Anti-NKp46 staining in 8 primary skin melanoma microenvironment (Red-brown cells) (A-H). Original magnification: ×10 (left panel) and ×20 (right panel) followed by computer magnification. (TIFF) [file pone.0076928.s002.tiff]
